# A qualitative study of influences on older women’s practitioner choices for back pain care

**DOI:** 10.1186/1472-6963-14-131

**Published:** 2014-03-21

**Authors:** Emma R Kirby, Alex F Broom, Jon Adams, David W Sibbritt, Kathryn M Refshauge

**Affiliations:** 1 School of Social Science, University of Queensland, St Lucia, QLD 4072, Australia; 2 Faculty of Health, University of Technology Sydney, Ultimo, NSW 2007, Australia; 3 Faculty of Health, University of Sydney, Lidcome, NSW 2141, Australia

**Keywords:** Back pain, Women, Qualitative, Interviews, Australia

## Abstract

**Background:**

Back pain is an increasingly prevalent health concern amongst Australian women for which a wide range of treatment options are available, offered by biomedical, allied health and complementary and alternative medicine (CAM) providers. Although there is an emerging literature on patterns of provider utilisation, less is known about the reasons why women with back pain select their chosen practitioner. In this paper we explore the influences on back pain sufferers’ decision-making about treatment seeking with practitioners for their most recent episode of back pain.

**Methods:**

Drawing on 50 semi-structured interviews with women aged 60–65 years from the Australian Longitudinal Study on Women’s Health (ALSWH) who have chronic back pain, we focus on the factors which influence their choice of practitioner. Analysis followed a framework approach to qualitative content analysis, augmented by NVivo 9 qualitative data analysis software. Key themes were identified and tested for rigour through inter-rater reliability and constant comparison.

**Results:**

The women identified four predominant influences on their choice of practitioner for back pain: familiarity with treatment or experiences with individual practitioners; recommendations from social networks; geographical proximity of practitioners; and, qualifications and credentials of practitioners. The therapeutic approach or evidence-base of the practices being utilised was not reported by the women as central to their back pain treatment decision making.

**Conclusions:**

Choice of practitioner appears to be unrelated to the therapeutic approaches, treatment practices or the scientific basis of therapeutic practices. Moreover, anecdotal lay reports of effectiveness and the ‘treatment experience’ may be more influential than formal qualifications in guiding women’s choice of practitioner for their back pain. Further work is needed on the interpersonal, collective and subjective underpinnings of practitioner choice, particularly over time, in order to better understand why women utilise certain practitioners for back pain.

## Background

Back pain represents a significant public health issue, affecting approximately 70% of the population over the life course [1-4], and incurring direct and indirect costs of greater than AU$8 billion per year in Australia [5-7]. Chronic back pain can be particularly debilitating, limiting capacity for work and participation in leisure and family life [8-12]. Given the rapidly ageing populations in Australia and OECD countries more broadly, the cost, provision and organisation of back pain care is a prominent healthcare problem [3,10,13,14]. The underlying causes of back pain are often difficult to identify, and treatment options are multifaceted and distributed across practitioner groups [1,15,16]. People with acute back pain who have sought treatment may experience slow or limited recovery [12], and progress relatively quickly from acute to chronic back pain status. In Australia, back pain care in its various forms includes general practitioners (GPs), medical specialists (e.g. rheumatologists), allied health providers (e.g. physiotherapists) and complementary and alternative medicine (CAM) practitioners (e.g. chiropractors, acupuncturists and massage therapists). The national publicly funded Medicare system reimburses patients (at least partially) for the majority of consultations and treatments provided by biomedical practitioners and some provided by allied health practitioners, but relatively few provided by CAM providers. In the case of CAM practitioners, the consultation and treatment is either funded out-of-pocket or is subsidised by private health insurance [17]. Despite these funding variations, the majority of chronic pain sufferers continue to seek care from multiple practitioners [1,18-20].

### Multiplicity in care options for back pain: choice and complexity

Biomedical, allied health and CAM practitioners offer a diverse range of treatments and approaches to acute and chronic back pain. While there is some cross-over between these areas of practice, they remain meaningfully distinct as provider groups. The degree to which practitioner care is evidence-based is also variable and inconclusive - both in terms of views on what constitutes evidence and level of evidence available [21-27]. As such, back pain care remains a contested territory of healthcare delivery. Existing work on back pain care has revealed some of the factors which may influence a patient’s choice of treatment. Studies in Australia have shown distinct patterns of referral for patients with back pain by general practitioners [28,29]. Patient expectations or outcome perceptions have also been shown to be significant. A UK study on primary care patients’ perceptions of lower back pain revealed an association between expectations and clinical outcomes, with poorer outcomes more likely for those who hold more pessimistic or ‘weak’ beliefs about expedient or controlled recovery [30]. Moreover, a systematic review of qualitative and quantitative studies on back pain treatment expectations found a persistent gap between the expectations of patients and the care offered by providers, highlighting the need for better strategies for meeting patients’ expectations around pain relief and the treatment experience (including communication, legitimization and confirmation of pain) [31]. A mixed methods study of chronic low back pain interactions with specialists in Norway found that ‘being taken seriously’ by practitioners was a key factor in patients’ accounts of the interpersonal experience of treatment [32]. Studies have also highlighted the need for practitioners to appropriately manage the emotional distress experienced by back pain sufferers [33-35].

Patient knowledge of the nature and availability of care options has been shown to be variable, and may be reflected in patterns of usage [15,20]. While the wide range of options for back pain care may enhance a sense of choice, it also presents potential for fragmentation of care, miscommunication and confusion. Moreover, access to biomedical, allied health and CAM choices is influenced by age, socio-economic status, geographical location, and education [3,17,36-38]. Patient utilisation of health care providers, particularly in the case of chronic illness, has been shown to be complex, where patients may choose to explore a range of modalities in complement, conjunction with, or as alternative to others [1,2,19,39-42]. While a growing body of work has focused on the potential benefits of collaborative approaches to care [43,44], we still know little about why patients choose one provider group or practitioner over another.

### Examining choice through the experiences of older women

Existing research illustrates that there are age and gendered dimensions to the uptake of back pain care. Women, for example, seek help for back pain (whether biomedical, allied health and CAM) at higher rates than men [3,36]. Studies have also shown that CAM has a greater presence in the context of chronic pain amongst older people [13,36,45-47], a product of an enhanced interest in wider therapeutic options at a time of general declining health [36]. Chronic back pain also represents a context in which choices may be shaped by gender and age. Studies internationally have explored various aspects of back pain care seeking behaviour, focusing on for example, practitioner usage [3,48], beliefs around pain and care [33,49], perceived benefits of CAM for back pain [50], and perceptions of care [20,32]. Yet, little is known about the ways by which decisions are made to approach a provider, particularly across the range of biomedical, allied health and CAM options. Qualitative examinations of subjective and individual experiences have complemented and balanced more population-based analyses of decision making and health utilisation [51-53]. This has involved exploring the subjective and community-based influences on provider choices as well as the role of lay knowledge and networks [54-56] on healthcare experiences and utilisation of practitioners across biomedicine, allied health and CAM. Yet such analyses have not been extended to women’s experiences of chronic pain and their negotiation of biomedical, allied health and CAM treatment options. In response to these gaps in knowledge, this study is focused on exploring the various influences on decision-making around treatment options, specifically to highlight any important factors outside of and separate from issues of clinical efficacy and effectiveness in going some way to explaining why back pain sufferers choose certain treatment options over others. The consumer and self-care movement has emphasised a focus on lay understandings and their role in mediating pathways through illness and care. Unlike other data collection techniques which can be limited, qualitative research which draws on, and interrogates, individual experience, can offer rich and detailed accounts of living and coping with back pain, and how consumers (in this case women) access and negotiate back pain care options.

## Methods

This project was conducted as part of the Australian Longitudinal Study on Women’s Health (ALSWH), designed to examine multiple factors affecting the health and well-being of Australian women over a 20-year period. The baseline cohort was recruited in 1996, and consisted of women in three age groups (18–23, 45–50, and 70–75 years) randomly selected from the national Medicare database [57]. The cohort has been shown to be broadly representative of the national population of women in the targeted age groups [58]. The focus of the current study is on the ‘mid-age’ cohort, aged 60–65 at the time of data collection, and was designed to explore women’s experiences of back pain, consultation practices, health service utilisation, attitudes towards treatment options and health status more broadly. For this study, women who had indicated that they had sought help from a healthcare practitioner for their back pain were recruited to initially complete a questionnaire [19]. During 2011/2012, and following University of Queensland ethics approval, a nationally representative sample of 1,310 women aged 60–65 completed a questionnaire (response rate: 80.9%) [19]. In the questionnaire, the women indicated a willingness to participate in a face to face interview (n = 513; response rate: 39.1%). Seventy-five women in South East Queensland indicated a willingness to participate, sixty-one were contacted and invited to participate (via telephone call), and fifty were interviewed. The interviews lasted for between forty-five minutes and two hours, and were conducted in the participants’ homes at a time of their convenience. The sample for the qualitative study included representation of a range of cases according to duration of pain, intensity of pain, urban/regional location, and employment status (as self-reported in the survey). The qualitative, semi-structured interviews continued until the point of data saturation. Of the sample of 50 women, 29 were in paid employment, 32 were married, and 43 had one or more children. The length of time with back pain broadly reflected patterns for the full questionnaire cohort (less than one year, n = 2; 1–10 years, n = 12; 11–20 years, n = 13; 21–20 years, n = 9; 31–40 years, n = 11; more than 40 years, n = 3). The mean length of time with back pain for the qualitative sample (n = 50) and questionnaire sample (n = 1,310) were 20.3 years and 20.4 years respectively. Interviews were audio recorded and transcribed verbatim, and were guided by an interview schedule (See Additional file 1) which was adapted to the individual circumstances of the interviewee. The interviews were designed to explore the following domains: decision-making practices and experiences of care; understandings of lay-professional and inter-professional communication; and, the meanings associated with various models of care.

### Data Analysis

Interview transcripts were entered into NVivo 9 software, which was then used to conduct a systematic thematic content analysis of the data employing a framework approach, according to the following steps: 1) Familiarisation - in which the researchers reviewed the manuscripts; 2) Identification of framework - key themes and issues identified around which the data was organised; 3) Indexing - application of themes to text; 4) Charting - use of headings and sub-headings to build up a picture of the data as a whole; and 5) Mapping and interpretation - in which associations were clarified and explanations worked towards [59]. Members of the research team provided initial independent coding, which was subsequently cross-checked in order to develop themes as we moved towards an overall interpretation of the data. Analytic rigour was enhanced by searching for negative, atypical and conflicting or contradicting cases in code and theme development [59-61].

## Results

Here we report the influences on women’s decision-making around practitioner and provider choice of treatment for their most recent episode of back pain. We refer to ‘providers’ and ‘practitioners’ respectively to distinguish between participants’ accounts of a group of professionals/practitioners (e.g. general practitioners, chiropractors or physiotherapists in general), and individual practitioners (e.g. an individual or named GP, chiropractor, etc.). Each of the women offered a detailed account of the practitioners they had visited or were planning to visit, and the circumstances which led to these decisions. All of the participants had sought care from at least one practitioner during their time with back pain. Four significant themes emerged from the interviews related to the influences on decision-making regarding health care service provider consultation: a) familiarity with treatment, and experiences with providers/practitioners; b) recommendations or anecdotes from networks of family/friends; c) proximity of practitioners; and, d) qualifications and credentials of practitioners. A summary of key findings and examples is included in Table 1.

**Table 1:** Summary of themes with examples

**Influences on health service utilisation**	**Example**
Familiarity/relationship with providers and familiarity with treatment type	- Current/previous experience with a provider group for back pain related or unrelated complaint
	- Familiarity with the treatment procedures for a provider group (irrespective or previous effectiveness for same or different ailment/complaint)
	- Exclusion of future encounters with a provider group due to previous negative experience
Family/social networks:	- Anecdotes from family/social networks for the effectiveness of a specific practitioner
	- Recommendations for a provider group
	- Recommendation based on second-hand knowledge or community reputation of a specific practitioner
Proximity of practitioners	- Attractiveness of nearby/local practitioners
	- Difficulty travelling to practitioners for treatment due to physical pain/mobility
	- Difficulties finding appropriate/suitable practitioners in local proximity
	- Highly valued practitioners worthy of travelling greater distances for treatment
Qualifications and credentials	- Research on internet/talking to friends/family about provider group approaches to treatment, efficacy and evidence base
	- Consideration/assessment of formal qualifications
	- Difficultly knowing what qualifications mean
	- Experience and practical expertise outweigh formal qualifications

### Familiarity with treatment type and of existing relationship with providers

Familiarity with the treatment techniques of a provider group, or a previous experience with an individual practitioner was a consistent theme, and was mentioned by all participants as a significant influence on their choice of provider for treatment. All participants had experienced visiting a GP (although not all for back pain related complaints), and three quarters of participants had visited at least one CAM or Allied Health practitioner (again, not universally for back pain-related complaints). The participant cohort had experienced a diverse range of providers: GPs, medical specialists, exercise physiologists, occupational therapists, physiotherapists, chiropractors, osteopaths, massage therapists, Bowen massage therapists, acupuncturists, Chinese herbalists, naturopaths, yoga and Pilates instructors, and reiki therapists. All participants had visited practitioners from more than one provider group for their back pain, and in the vast majority of cases, the knowledge gained from such experiences was talked about as a significant influence on current and future decision-making. Although prior encounters with practitioners were by no means unanimously positive, the familiarity afforded by these experiences remained important. That is, a prior experience, whether talked about as positive or negative (in terms of the effectiveness or success of the treatment, satisfaction, or the interpersonal encounter), enabled the participant to retain some level of familiarity with and understanding of the style of treatment offered. In an area of perceived significant diversity, chronic pain was viewed by the participants as a particularly difficult issue to navigate, and thus familiarity with a practitioner or with a therapeutic approach was key to decision-making. Those participants who benefited from or recalled positive experiences regarding a previous encounter with a practitioner in all but two cases said that they would seek (or had already sought) care from a practitioner from the same provider group.

In circumstances where previous experiences were for conditions which were reported as removed from, or not relevant to, back pain care (for example, rehabilitation for a broken ankle, or treatment for diabetes), the majority of participants maintained loyalty to the respective provider group. This loyalty with the specific forms of treatment was justified in two ways: either in terms of the attractiveness of a particular practitioner approach, or the perceived ‘evidence base’ of the provider groups’ techniques. There were many examples in the interviews of highly regarded and recommended individual practitioners, as well as frequent examples of the experience of a provider group’s approach or methods of treatment serving to legitimize that approach. However, nine participants cited negative experiences with a practitioner (for example a dislike of the treatment, or a lack of improvement following treatment) as justification for avoiding the associated provider group in the future. Overall, the experience of attending a practitioner enabled a familiarity with the techniques employed, adding to the perception of the provider group as a credible and attractive option for back pain care. Ultimately, the effectiveness of a previous treatment was, broadly speaking, less relevant to the current decision-making process than the knowledge and understanding of the treatment techniques and interpersonal approaches gained by these experiences. Examples of indicative quotes related to the influence of familiarity with treatment style and of existing relationships with providers are provided in Table 2.

**Table 2:** Indicative quotations: Familiarity/relationship with providers and familiarity with treatment type

**Participant**	**Example**
Interviewer:	So why [did you choose] chiropractic?
#16	Why? Because I had had chiropractic before… for my neck actually, because I’ve done a lot of office work, and I think bad posture, sitting over computers and all things like that, for my neck, and I think just tension in there, and they managed to relieve that. So ah, that was where I found ah, and I just had a confidence there, I had a confidence and I felt it was helping.
#8	So I already knew somebody [a physio], [who runs my] hydrotherapy class. So I organised…to do the hydrotherapy in the morning. And the lady that does it is a physio, and I also arranged to start going to her as well…because I knew that, I’d been already previously.
#4	But I had a really bad experience with one person where I thought, no, never again, it was really painful and horrible.
#25	Like I had [a bad] experience with [a] chiropractor where initially I was quite happy, and then I wasn't. But that's not going to stop me going back if someone says to me, “I have a really good chiropractor, why don’t you just try him?” I’d go.
#30	We’re very happy with our GP, we’ve been with her for quite a number of years, and she keeps an eye on us. We go every six weeks.

### Family/social networks

A second key theme identified from the analysis of the interviews was the role of family and/or social networks in informing participants’ decision-making about health care providers. Multiple examples were offered by the participants about recommendations of both provider groups (e.g. “my sister told me I should see a physiotherapist”) and individual practitioners (e.g. “She [best friend] put me in touch with [acupuncturist], who she swears by”). The example quotations in Table 3 indicate the significance of the opinions of family and friends, as well as the important influence of wider social networks including anecdotes from neighbors or work colleagues. Each of the fifty participants talked about their back pain care options as a topic for which they had sought advice from those around them, and within the interviews there was frequent reference to decisions based on a recommendation from social networks. For some interviewees, recommendations were based on personal experiences with a particular practitioner, while for others the recommendations were for a provider group, rather than a specific practitioner. The prevalence of back pain within the wider community was also evident in such accounts. That is, each knew of a family member, friend, neighbor or work colleague who suffered from some form of chronic back pain. Thus, for these participants, talking about their back pain care options and seeking recommendations was viewed as straightforward and a logical step in the decision-making process.

**Table 3:** Indicative quotations: Family/Social Networks: Recommendations and anecdotes

**Participant**	**Example**
#19	Because people had said…that this was a good thing to try.
#10	Yeah. And the guy over the road said that he went to a [practitioner], who is up over here at [local suburb], and said “he’s really good.”
#6	Invariably it would've been a friend’s referral that might have done it.
#12	I thought “yes, I’ll make an appointment,” and I went to the [practitioner] that my girlfriend goes to and swears by…
#32	But you always ask people as well who they recommend, a personal recommendation is better than any advert.
#33	…I think hearsay is an important one, where someone's been and they’ve given relief.
#36	Well, I usually talk to people, especially if I move to a new place where I don’t know anyone, I’ll talk to people, and say “well where do you go?” or “what do you do?”

### Proximity of practitioners

A third key theme from the interviews was the proximity of practitioners when finding an appropriate provider of back pain care. This element of convenience, while often inextricably linked to the recommendations of friends, family or colleagues, also operated beyond such networks. The logistics of accessing providers (the availability of providers local to, or in close proximity to, participants) was an initial consideration. Convenient access to a practitioner was cited frequently by participants who were mindful of the physical pain incurred by travelling sometimes even only short distances for treatment. Thus, participants often used the local telephone directory or the internet to seek out providers nearest to their homes or work. In fact, for 12 participants, their choice of health care provider (both practitioner and associated provider group) was talked about as solely dictated by locale. One participant, for example, said: “I got in the car and drove until I saw some place that was open…I’d never been to a chiropractor before, I just happened to find somewhere that was open that I figured might help”. Interview transcripts also revealed numerous accounts of provider usage based on local advertising within the participant’s suburb of residence, with decisions about practitioners often occurring as a result of conversations about nearby providers with friends and family members. The convenience of local practitioners was frequently revealed by the anxiety and disappointment talked about by more than half of participants when recounting experiences of a valued practitioner relocating. Circumstances where a participant had attempted to contact a practitioner for treatment for their most recent episode of pain, only to discover that the practitioner had relocated to elsewhere within the area (or state/interstate), were common. The relocation of practitioners represented a difficult choice for participants; that is, to travel further to visit a familiar and trusted practitioner, or to seek a ‘new’ practitioner closer to their home. The task of finding another local practitioner was talked about as arduous, but essential, as the convenience of having a nearby (but as yet unknown) practitioner often outweighed travelling further to a known and appreciated practitioner. However, (and indicative of their highly valued relationships with specific practitioners) we note here that for five participants, mobility and pain were necessary albeit unfortunate prices to pay in order to visit a “treasured” practitioner whose practice was located further afield. Examples of indicative quotes related to proximity of practitioners are provided in Table 4.

**Table 4:** Indicative quotations: Proximity of practitioners

**Participant**	**Example**
#47	I'm trying to negotiate, or try and meet some new people [practitioners] up here in [town], so I don’t have to travel so far.
#15	I’m [currently] trying to find somewhere [any practitioner] nearby…
#3	I thought, “well what am I going to do?” So I looked up, and I found this person, and this was really off-the-cuff, who was close by at [suburb] where we lived at the time…
#27	But normally, it is word of mouth, or the yellow pages, or the Internet, its hit and miss.
	I might look up, depending how quickly I want to get there, look up the Yellow Pages, what I usually do. Sometimes I’ll ring a couple of them and just ask a few questions.
#18	So I thought “well, nothing ventured nothing gained,” so I gave a ring, gave a call, and it turns out that he just lives down the road from me here, and that's where he practices from. So I just think “well, give it a try.”
#28	Just picked him out of the book, the phonebook I think.

### Credentials and measures of validity

The final key theme revealed by our analysis was the significance of practitioner’s qualifications and credentials, specifically how expertise was assessed when choosing a practitioner for back pain care. Information on the internet, or to a lesser extent, from literature provided by GPs or friends/colleagues, was reported to be used by half of the participants to learn about particular treatments that were less popular or not well understood. Such information was also utilized to seek out information on individual practitioners through for example, existing reviews by previous or other current patients. Participants who engaged with such background research all talked about uncovering information as a key part of the process of locating and choosing a practitioner, and viewed the process of finding out about service provider groups and individual practitioners as good practice. That is, identifying as a conscientious and informed patient was central to their experience of pain and help-seeking behavior, and also allayed some of the fears associated with popularized notions of “unsafe” or “quack” practitioners. Thus, by engaging in research and attempting to assess a practitioner’s qualifications or credentials, a patient may feel more secure that seeking treatment from their eventual choice of practitioner will be more worthwhile.

It is important here to note the difficulties many participants experienced in measuring or assessing a practitioner’s qualifications. The interviews contained frequent reference to a lack of lay-understanding of the meanings or indeed practical implications of a range of qualifications. This was evident when talking about biomedical, allied health and CAM providers, where there was little understanding of how to assess the gravitas of a variety of credentials. A practitioner’s credentials were talked about as difficult to “translate”, particularly when listed as acronyms following their name, or when referencing a governing body or membership to a professional organisation that was unfamiliar. So, while some participants talked about being “impressed” by formal qualifications, others were more dismissive of practitioners’ formal qualifications because of the difficulties for patients in knowing if or to what extent these were recognized and by whom. Moreover, a practitioner’s formal qualifications were rejected by some participants in terms of the relevance of such credentials to the quality or potential outcome and experience of the treatment offered. Half of the participants talked about the lack of necessary correlation between formal qualifications and the overall credibility of the practitioner in terms of treatment success - the experience of the treatment, the relationship with the practitioner, and the physical and psychological relief provided (or imagined as potentially provided) outweighed the importance of formal qualifications. Examples of indicative quotes relating to the influence of credentials and measures of validity are provided in Table 5.

**Table 5:** Indicative quotations: Qualifications and Credentials

**Participant**	**Example**
#3	And I, as far as I can, I make sure that they’re registered with whatever body is relevant to that particular practice… Online, and researching it that way. And I know that there is a list of physiotherapists who are trained in dry needling, and that’s the term that they use on the Internet, so it’s all listed there, they even have the qualifications that they have done, whether it’s the introductory course, whether its lower sections or upper sections of the body, it’s very specific.
#19	Oh, I don’t think that qualifications are particularly helpful, it’s only by experience with them and working with them that you start to work out whether they’ve got the goods or not
#12	It impressed me with the initials after her name because there are a lot of people around that don’t have the qualifications, and you’ve got to be very careful about that, because they can do you lot of damage as well.
#18	I am conscious that they do need to be qualified, not just some person that decides to put their name and practice in something. So you know, the qualifications is probably part of the judgement you sort of make, yeah.
#25	And people don’t necessarily have to have the high qualifications to be really good at what they do you know, some people just have that natural affinity for touch, and they can just do it, they’re brilliant. And I'm open to all of that.
#35	Now with chiropractic, that's a little bit easier in that you can see their degree, you can see a chiropractic degree, and in this case with my current bloke, I can see he's got his degree and he’s done his Masters in something else, and Masters in something else, and is doing one in public health at the moment. And I think “well, he's not an idiot,” and “he’s not someone who’s gone and printed something off the computer for you know, a certificate, that doesn't mean anything, and I can see that it came from which university. So I like to see that up first. With Bowen or massage or something, that's a little bit trickier…I think I saw her certificates and stuff up on the wall, but really honestly, that doesn't mean anything to someone like me. I can't, that you know, if the Bowen Therapists Association or something or other has issued a certificate, it probably doesn't mean much to anyone who isn't already doing Bowen maybe you know, if you're not involved in it already, you probably won't know whether that means much or not.

## Discussion

Back pain care is a highly diversified area of health service delivery and the majority of sufferers actively juggle multiple treatment and provider options concurrently or over time. In order to do so, and as illustrated in the results of the current study, sufferers draw on, and are influenced by, various stakeholders, social networks and resource practicalities. Although formalised (largely biomedical and allied health) models exist which aim to provide and deliver appropriate and evidence-based care to chronic back pain sufferers, the results presented here suggest that women seek help beyond biomedical and allied health practices, and furthermore, that subjective, interpersonal and social factors fundamentally shape their choices and actions. While the production and existence of a biomedical evidence-base may drive funding and delivery of many back pain care practices (at least those provided via the State), from the perspective of individual sufferers, familiarity, convenience and resonance with social networks may in fact be more significant in their decision-making about practitioners.

A key process identified here was the lay consultation process which was focused on establishing appropriate provider types and recommendation for a specific practitioner. Once a decision to seek some form of treatment had been made, recommendations from social networks, familiarity with provider-specific treatments and proximity to practitioners were the main drivers of action. Our findings reflect the diversified and often fragmented landscape of back pain care. Moreover, our analysis reveals that back pain sufferers lack guidance (in the form of clinical evidence) for choices around care seeking. The lack of coherent and systematic support or clinical evidence across biomedicine, allied health and CAM in the context of back pain [21-27] may create a decision-making context which is heavily mediated by lay knowledge and expertise. In our study it was clear that participants wanted to know more about available treatment options, but found such information difficult to access and/or assess or measure. Participants thus sought social and community-based avenues for advice to manage their own care pathways [56,62]. In such circumstances, trust may become more focused on lay sites of expertise rather than on practitioner and formalised expertise. In line with existing research, our findings show an explorative approach to care, and a willingness to try various treatment options (biomedical, allied, and CAM) [15,19,39]. Moreover, our findings add to the emerging body of work which has drawn attention to the significance of treatment for back pain as an interpersonal and interactional experience [31,32,34,63].

Ultimately, the interviews illustrate that within the context of a pluralistic and often fragmented system, involving significant self-management, there may be key distinctions between professional and lay foci on care. Whilst providers may be focusing on evidence-creation in their own area of practice, in the community issues around access, familiarity, relationships, social networks and confidence are key drivers of choice and care pathways. Research into back pain care and choice has largely focused on beliefs, perceptions and preferences around care [20,32,33,49,63], and rarely have there been rigorous qualitative examinations of the factors which underpin illness experience and practitioner engagement. While clinical research has focused on what practitioners do and outcomes (whether subjective or objective), here we have focused on what leads women to engage practitioners. Put simply, our findings raise questions about the importance of what practitioners actually ‘do’ for people looking for available back pain treatment, compared to practitioner personality, ‘track record’, reputation and standing within the community. While these facets clearly work in tandem, previously we have seen insufficient emphasis on, and exploration of, the pathways to care and the assumptive basis of patient action.

This study has various limitations. Although our sample of women is relatively large for a qualitative study, it only captures the experiences of women from one geographic region, and from a specific age group. As such, our findings cannot be generalised to other people in other settings, nor to those of the broader nationally representative sample from which participants in this study were drawn. Furthermore, the sample of women included in the study all experienced chronic back pain, and for a significant number of years. Our findings therefore may not represent the views or experiences of people with acute back pain, or those who have not lived with (often unsuccessfully treated) back pain for a considerable period of time.

## Conclusions

This study provides a means for better understanding the lay underpinnings of choice and action in the context of chronic back pain. Given our findings, further research is needed on the social and community influences on (and determinants of) practitioner engagement and the development of models which capture clinical efficacy and lay experiences concurrently. An absence of such understandings will further marginalise decision-making practices from practitioners (who are providing or potentially providing care), leading to sub-optimal communication, teamwork and coordination of care across sectors, as well as having negative impacts upon recovery.

## Abbreviations

ALSWH: Australian Longitudinal Study on Women’s Health; GP: General Practitioner; CAM: complementary and alternative medicine.

## Competing interests

The authors declare that they have no competing interests.

## Authors’ contributions

AB, DS and JA conceived of the study, and supervised the data collection. AB, EK, DS, JA and KR participated in the design of the study. EK conducted the data collection. All authors were involved in and contributed to the data analysis. All authors participated in drafting and revising the manuscript, and all authors approved the final manuscript.

## Pre-publication history

The pre-publication history for this paper can be accessed here:

http://www.biomedcentral.com/1472-6963/14/131/prepub

## Supplementary Material

Additional file 1 Interview schedule. Click here for file
