# Supplementary material for: A qualitative study of influences on older women’s practitioner choices for back pain care
Source: BMC Health Serv Res. 2014 Mar 21;14:131. doi: 10.1186/1472-6963-14-131 (PMC3998023; doi:10.1186/1472-6963-14-131)
Supplement: Additional file 1 — Interview schedule. [file 1472-6963-14-131-S1.docx]

**Interview Guide for Women with Back Pain**

**Indicative questions for semi-structured interviews**

1. *Background*
   1. Could you please tell me about your most recent experience of back pain?
2. *Women’s decision-making practices and experiences of care*
   1. What different types of practitioners have you sought help from?
   2. What has your experience been of the different types of practitioners?
   3. Where did you get information about treatment for back pain?
   4. How did you make decisions about who you were going to see during your most recent experience of back pain?
   5. How did you make decisions about what is safe or risky to treat your back pain?
   6. How did your expectations for the treatment sought for your most recent back pain relate to your actual experience?
   7. Why did you consult the practitioners/types of care you chose?
   8. In terms of the different health professionals that treated your back pain, did you notice any differences or similarities in their approach to care?
3. *Understanding lay-professional and inter-professional communication*
   1. Could you please tell me about your interactions with your health care practitioners?
   2. Could you please tell me about the communication styles of your various practitioners and to what extent did they have similarities or differences?
   3. How did they represent other professions when talking to you?
   4. Were you aware of any communication between your health care providers? How was this received?
4. *Living with back pain*
   1. Could you please tell me about what life is like for you living with back pain?
   2. What have been your experiences with family, friends, colleagues in talking about/understanding your back pain and back pain care?
   3. In what ways and to what extent does your back pain affect your day-to-day life?
   4. In what ways do you manage your back pain on a day-to-day basis?
   5. Could you please tell me about the things you do to manage your back pain yourself?
